# Use of a Simple Pain Assessment Tool in a Shared Decision-Making Approach to Tapering Chronic, High-Dose Opioids

**DOI:** 10.7759/cureus.18892

**Published:** 2021-10-19

**Authors:** Sonya Del Tredici, Anthony Iacoviello

**Affiliations:** 1 General Internal Medicine, WellSpan Health, York, USA; 2 Internal Medicine, Drexel University College of Medicine, Philadelphia, USA

**Keywords:** pain assessment, pain, shared decision-making, chronic opioids, taper, opioid epidemic

## Abstract

Because of the opioid overdose epidemic, over the past decade, efforts have been made to reduce opioid prescribing. However, tapering patients off their chronic opioid medications can lead to opioid withdrawal and increased chronic pain, use of illicit opioids, mental health crisis, and opioid overdose. Strategies for safely tapering opioids are needed because of these risks. This case report describes a 57-year-old male suffering from chronic back pain who had been on high-dose opioids for over 20 years. Using shared decision-making, a simple pain assessment tool, and a patient-guided taper schedule, he was able to taper off his opioids completely. This case highlights a successful opioid taper and presents simple strategies that can be used by patients and clinicians who are working to reduce chronic high-dose opioid use.

## Introduction

From 1999 to 2019, there has been a steady increase in drug-related overdose deaths in the United States. Opioids have been responsible for the majority of overdose deaths, causing 49,860 deaths in 2019 [1]. The COVID-19 pandemic has worsened the crisis, and preliminary data show a 29.4% increase in drug overdose deaths from 2019 to 2020, with 69,710 of those deaths involving opioids [2].

Prescription opioids have been a major contributor to the opioid epidemic. At the peak of opioid prescribing in 2012, there were 225,207,954 prescriptions for opioids dispensed [3]. Roughly 21%-29% of patients receiving opioids for chronic pain misuse them [4], and the risk of opioid overdose death is directly related to the prescribed daily dose of opioid medication [5]. Furthermore, it is estimated that 4%-6% of those who misuse prescription opioids eventually progress to heroin, and about 80% of people who use heroin first misused prescription opioids [6]. 

Fortunately, the rate of opioid prescriptions has been declining after reaching a zenith in 2012 [3], driven in part by the 2016 recommendations from the Center for Disease Control (CDC) and Veterans Affairs (VA). The CDC defines high-dose opioid prescriptions as ≥90 morphine milligram equivalents/day (MME/day), with the VA then recommending that patients meeting this criterion be evaluated for opioid dose reduction [7,8]. However, tapering opioids is not without risk to patients. Recent data demonstrate that for patients on stable, long-term, higher-dose opioid therapy, tapering was associated with an increased risk of overdose and mental health crisis [9]. Patients are also at risk for opioid withdrawal, increased pain, and transition to illicit opioids. In this case report, we describe a shared decision-making strategy for tapering stable, long-term, high-dose opioids using a simple pain assessment tool.

## Case presentation

The patient provided consent for the writing of this case report and assisted in the summary of his story. He is a 57-year-old male who presented for a new primary care visit to address his back pain. He had a 35-year history of chronic back pain, initially caused by two motor vehicle accidents and exacerbated by a physically demanding factory job. Over the decades since his initial injury, his pain had worsened, and he became progressively disabled, eventually unable to work and limited in many activities. He had attempted to find relief through surgery, including a discectomy and laminectomy with an L5-S1 fusion, nerve ablations, spinal injections, physical therapy, massage therapy, chiropractic treatments, home exercises, and many different medications. He had been on chronic high-dose opioids for over 20 years.

His pain was severe, limiting his activities on most days of the month. He had diffuse pain from his neck down to his lumbar spine and pain, numbness, and tingling radiating to his bilateral groin and down his left leg. MRI of the lumbar spine done several months prior to his initial visit revealed multilevel severe degenerative changes, but no significant nerve impingement.

The patient was on a stable regimen of medications, including morphine ER 120 mg daily, pregabalin 75 mg three times daily, naproxen 440 mg twice daily, diclofenac 75 mg twice daily, tizanidine 8 mg at bedtime, and escitalopram 20 mg daily. He also took medication to treat hypertension, high cholesterol, constipation, and acid reflux. The patient’s pain management specialist had recently retired, and the patient had to travel over an hour to see a new pain management physician who would prescribe him opioids. This new physician had recently told him that he would have to reduce his morphine dose to 90 mg/day to comply with the 2016 CDC prescribing guidelines.

Tapering

The patient suffered several adverse effects of opioid therapy, including constipation, sedation, and depression. He was also becoming concerned about the safety of his medications and frustrated with the increasing difficulty of obtaining them. However, when he considered a taper, the patient had a significant fear of being “cut off” from his medication, losing access to his pain management physician, and having increased untreated pain. The PCP called the pain management physician, who agreed that the patient could return to his care and resume morphine 90 mg daily if the taper caused a significant increase in pain. Providing this safety net reduced the patient’s fear, and he agreed to try a taper, with an initial goal of 20-50 mg daily of morphine (the dose range associated with a lower risk of overdose [10]).

From his starting dose of 120 mg daily of morphine, the patient reduced his dose by 10 mg daily every month. After each decrease in dose, he would have several days of significant pain, and he feared the taper was worsening his overall pain and level of functioning. He was given a simple method to assess his progress and track his symptoms, developed by his PCP. He rated each day as either “good” or “bad” and defined for himself what defined “good” and “bad.” A “good day” was defined as a day when he was able to go to the gym, do errands and chores around the house, and visit his mother. A “bad day” was a day where he was confined to his recliner due to pain. 

**Figure 1:**
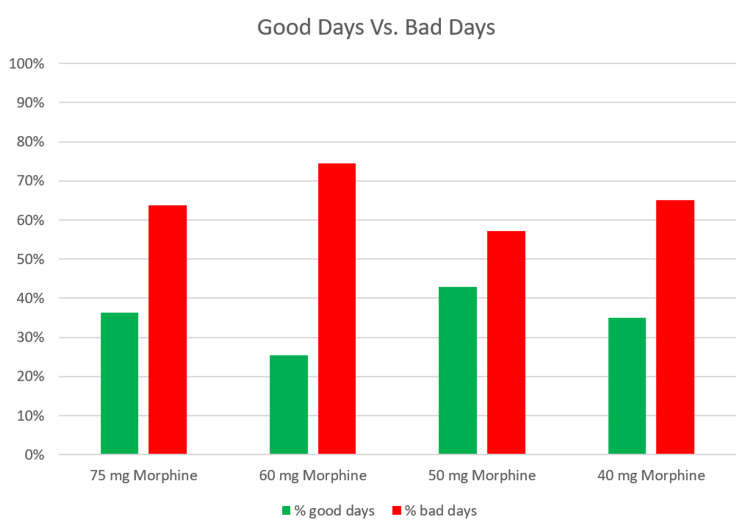
A reproduction of the patient-generated graph of pain data, showing the proportion of good days and bad days at different daily morphine doses during the opioid taper. The patient did not track consistently at other doses.

With this assessment tool, he was able to see that he had three to four “bad” days after each dose reduction and then a return to his regular level of functioning. He also noted the proportion of good and bad days at each morphine dose and created a graph of his personal pain data (see Figure 1). He saw that the proportion of “bad” days did not change but stayed at approximately 65% after each dose reduction and that reducing his opioid dose did not change his overall pain level. This gave him hope that he could continue the taper without worsening his chronic pain or diminishing his functioning.

Final outcome

Although the initial goal had been to reduce his morphine dose to below 50 mg daily, the patient continued tapering beyond that, and after 10 months, he was able to stop opioids altogether. Other than increased pain, he did not experience any symptoms of opioid withdrawal. He also did not change his other methods of managing his chronic pain, which at that time included exercise and various non-opioid medications. At the end of the taper, his back pain was unchanged, but he had significant improvement in opioid side effects, including constipation, sedation, depression, and disengagement with his family. Two years later, he remains opioid-free. He is very happy with the outcome and feels that tapering off the opioids has significantly improved his quality of life.

## Discussion

This case illustrates the effectiveness of a shared decision-making strategy for tapering chronic high-dose opioids. Key components of the strategy are using shared decision-making, alleviating the patient’s fear by providing a safety net in the case of worsening symptoms, using a simple daily pain assessment tool for the patient to track his own progress, and allowing the patient to determine the pace of the taper.

The shared decision-making was particularly important, including extensive discussions with the patient regarding the risks and benefits of tapering that focused on the impact of opioids on his life. This allowed the patient to choose tapering as the best option for him and helped him commit to the process. It is also important to recognize that a taper might have adverse outcomes and address this as part of the shared decision-making. While not all patients can be given the option to return to previous levels of opioid prescribing if the taper worsens pain or suffering, there are other ways to plan for potential adverse outcomes. The patient could be assured of slowing or pausing the taper in the case of increased pain, provided with low-dose opioid medications to take as needed to combat unexpected increased pain, offered other modalities to treat the pain, and most importantly assured that they will not be abandoned by the physician as they go through the process.

The simple pain assessment tool we developed was also an important aspect of our strategy. While many pain scales exist, ranging from simple 0-10 numeric scales to complex questionnaires with hundreds of options, they are not designed for daily home use and may not capture those aspects of the patient’s experience that are most important to them. Using our tool, the patient defined for himself what was a good day and what was a bad day, and the daily assessment was easy enough not to become burdensome.

While there is no common standard for the best rate of opioid tapering, expert opinion recommends slow tapers for patients who have been on opioids for a long time. The CDC and the Department of Health and Human Services (HHS) guidelines [7,11] recommend a taper of 10% per week to 10% per month, and we find that allowing the patient to guide the pace of the taper minimizes discomfort and fear. In this case, the patient chose to decrease his daily dose by 10 mg at a time for ease of dosing.

## Conclusions

Extensive education on safe opioid prescribing, guidelines from professional and governmental organizations, and the emerging data about the dangers of opioids have driven a significant reduction in prescribing over the past nine years. However, many “legacy” patients continue to be prescribed chronic high-dose opioids that were started decades prior. As they age, this group becomes more vulnerable to adverse outcomes from their opioids and risks losing access as fewer and fewer prescribers are willing to maintain patients on these medications. It is important to find safe strategies to taper these opioids when possible, focusing on the patients’ quality of life and supporting them throughout the process.
